# Transillumination-guided Study of the Endoscopic Anatomy of the Lacrimal Fossa

**DOI:** 10.1590/S1808-86942010000100007

**Published:** 2015-10-17

**Authors:** Luiz Artur Costa Ricardo, Marcio Nakanishi, Antonio Sérgio Fava

**Affiliations:** 1 MSc; MD; 2 PhD in Otorhinolaryngology - University of São Paulo Medical School; Assistant PhD - Department of Otorhinolaryngology - University of Brasilia Teaching Hospital; 3 PhD in Head and Neck Surgery - University of São Paulo Medical School. Chair of the Head and Neck Surgery Department - Hospital do Servidor P úblico Estadual, São Paulo

**Keywords:** lacrimal apparatus, dacryocystorhinostomy, transillumination

## Abstract

Dacryocystorhinostomy is the treatment of choice for the obstruction of the lachrymal apparatus. At the end of last century, the development of the endoscopic instruments for nasosinusal surgery has made it possible to do it through the endoscopic pathway. Nonetheless, anatomical variations make it difficult to have reproducibility endonasaly.

**Aim:**

study the endoscopic anatomy of the lachrymal fossa through transillumination of the common canaliculus.

**Study design:**

experimental.

**Materials and Methods:**

we dissected 40 lachrymal pathways from 20 human cadavers, in three stages: 1. identification and dilation of the lachrymal canaliculus. 2 -Optic fiber beam introduction; 3 - endoscopic dissection of the lachrymal sac, describing its position.

**Results:**

the most frequent position of the lachrymal sac was between the free border of the middle turbinate and its insertion immediately underneath it. The maxillary line was seen in 95% of the cases. Septoplasty was needed in 12.5%, unicifectomy in 35% and middle turbinectomy in 7.5%.

**Conclusion:**

Although the lachrymal sac has a more frequent location, its position varied considerably. The transillumination of the common canaliculus proved useful, solving the problem of the anatomical variability.

## INTRODUCTION

The nasolachrymal duct stenosis causes constant tearing (epiphora), recurrent dacryocystitis and mucocele formation. Dacryocystorhinostomy (DCR) is the surgical procedure of choice for the treatment of these conditions and it is based on creating a permanent communication canal between the lachrymal apparatus structures and the nasal cavity by means of a bony window (rhinostomy) opened through a nasal wall resection at the lachrymal fossa located on the lateral wall of the nasal cavity.

Dacryocystorhinostomy can be carried out externally (DCR-ex), or endonasal (DCR-en). The external approach was described by Toti^1^ and, with some modifications it was the standard procedure for the treatment of obstructed lachrymal pathway, always done by ophthalmologists. Before Toti, Caldwel^2^ had described the endonasal approach, advocating the bony resection on the lateral nasal wall through the inferior portion of the middle meatus. The development of endoscopic instruments for nasosinusal surgeries in the recent decades, with a better visualization of the intranasal structures has triggered again the interest of researchers and surgeons in approaching the lachrymal sac through the nose. Nonetheless, there are significant anatomical variations in the middle meatus regarding its conventional reference points (middle concha, uncinate process, ethmoidal bulla) and of these with the lachrymal pathway, making it difficult to have a reproducible technique to approach the lachrymal pathway through the lateral nasal wall based solely on anatomical parameters.

## OBJECTIVE

The present paper aims at using common canaliculus transillumination in order to study the surgical anatomy of the lachrymal fossa of the lateral nasal wall using the endoscope, analyzing its relation with the anatomical reference points of the nasal cavity, especially middle meatus and nasal septum structures, thus contributing to a standardization of the procedures during endoscopic dacryocystorhinostomy (DCR-en).

## METHODS

We studied 40 lachrymal pathways from 20 young adult cadavers (20 to 40 years of age), from both genders, without history of facial trauma, endonasal surgery approaching the lateral nasal wall or lachrymal pathway surgery. The dissections were done according to approval from the São Paulo Coroner's Office and approval from the Ethics in Research Committee from the Instituto de Assistência Médica ao Servidor Público Estadual (CEP/IAMSPE) under protocol # 070/03.

The cadavers were placed on the autopsy table, in horizontal dorsal decubitus, having their chest and heads covered in plastic with a triangular hole in the face in order to expose only the nose and the medial portion of the eyelid corners.

Afterwards the nasal cavities were cleaned with gauze soaked in water and chlorhexidine, followed by the use of dry pads in order to remove the excess of secretion that could be still present.

The study was based on three basic stages: 1 - lachrymal pathway identification and dilatation; 2 - introducing the optic fiber into the dilated lachrymal pathway and; 3 - endoscopic dissection of the lachrymal sac on the lateral nasal wall.

### Stage 1 - Lachrymal pathway identification and dilatation.

**Figure 1:**
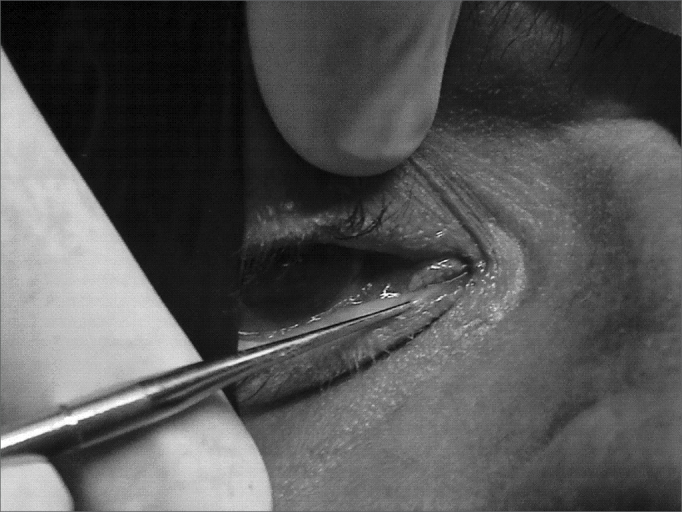
Introducing the dilator in the lachrymal point through the inferior canaliculus.

A # 1 Bowman probe was introduced into the lachrymal point of the upper or lower lachrymal canaliculus. The probe was first introduced vertically in the lachrymal point and the ampulla, and then rotated 90^o^ laterally in the same plane, according to the angle formed in the first portion of the canaliculus, and it was pushed forward until feeling a bony consistency, indicating that the nasal wall of the lachrymal sac was reached. The Bowman probe is then removed and, following that, the lachrymal point dilator is introduced in the same canaliculus, following the same route (Fig. 1).

### Stage 2 - Optic fiber introduction.

**Figure 2:**
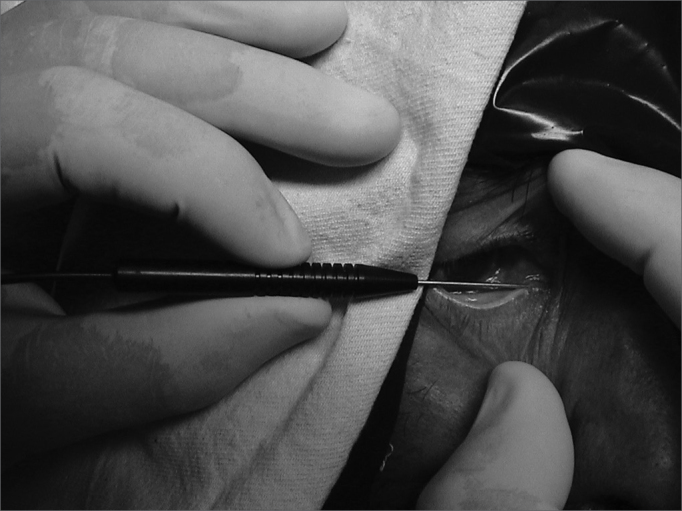
Introducing the optic fiber into the inferior lachrymal canaliculus.

The dilator is removed and we introduce the optic fiber in the dilated canaliculus, pushing the fiber all the way to the nasal wall of the lachrymal sac (Fig. 2).

The optic fiber is connected to an H. Osawa 150w light source, in order to transilluminate the contact point of the fiber with the bony wall of the lachrymal fossa, corresponding to the projection of the common canaliculus on the lateral nasal wall. This point is identified as the point of the greatest light intensity on the nasal wall and it is seen through the introduction of a rigid zero degree 4mm Endoview endoscope inside the nasal cavity.

### Stage 3 - Lachrymal sac dissection.

The lachrymal sac position was described in function of the transillumination point, as follows:

• Transillumination point position in relation to the middle turbinate insertion (coronal plane): above the insertion; immediately below the insertion, defined as a 5 mm distance from the turbinate insertion in the coronal plane; and below the insertion, when the point was located farther than 5 mm below the insertion in the same plane.
• Transillumination point position in relation to the middle turbinate head (axial point): anterior to the head; posterior to the head and anterior to the middle turbinate; and posterior to the head and middle turbinate insertion.

**Figure 3:**
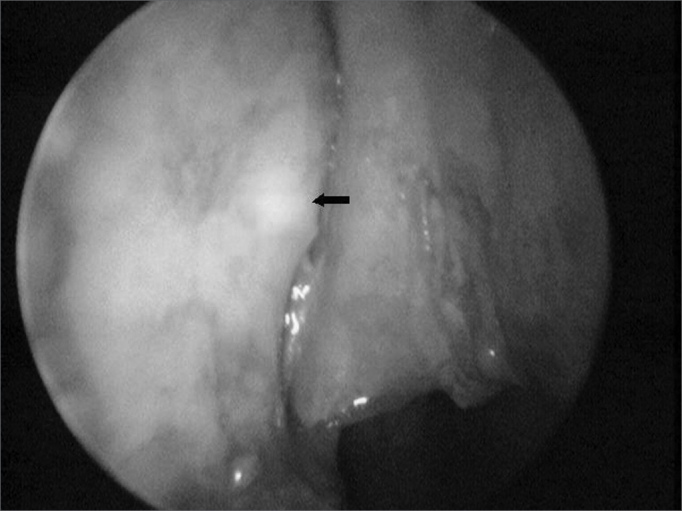
Transillumination point (arrow) on the bony lateral wall of the nasal cavity.

We made mucosal flaps on the lateral nasal wall of approximately 1.0 cm2 around the transillumination point (Fig. 3) from a vertical incision. After shifting it antero-posteriorly, the flaps were systematically removed, clearing the nasal wall. The lachrymal fossa was exposed posteriorly by the elevation of the lachrymal bone by using a Freer instrument in its anterior portion by removing the maxillary bone with a chisel and hammer.

Besides the transillumination point position, other anatomical parameters related to the endoscopic approach of the lachrymal pathway were described, as follows:

• Maxillary line: prominent or not;
• Nasal septum: evaluation as to the presence of septal deviation preventing surgical access to the lachrymal sac;
• Unciform process: evaluation as to the need or not of its removal;
• Middle turbinate: evaluation as to the need or not for its removal.

## RESULTS

A bilateral dissection was possible in all the 20 cadavers, as well as the visualization of the transillumination point on the lateral nasal wall, through the optic fiber introduced through one of the lachrymal canaliculi.

The transillumination point was seen in all the 40 lachrymal pathways studied and when we analyzed the axial plane there were three pre-established locations: anterior to the middle turbinate head, posterior to the middle turbinate head and anterior to the insertion, and posterior to the insertion; and when analyzed from the coronal plane, the locations were: above the middle turbinate insertion, immediately below the insertion - when found 5 mm below it, and below the insertion - distance greater than 5mm.

The transillumination points found were analyzed in terms of their relation to the intersections with the axial and coronal planes, with the intent of defining the lachrymal sac location on the lateral wall of the nose and its variations (Table).

The transillumination point, when located anterior to the middle turbinate head was above the insertion in none of the cases. In seven cases (17.5%) it was anterior to the turbinate head and immediately below the insertion and, in two cases (5%) it was anterior to the turbinate head and below the insertion.

The transillumination point, when located posterior to the middle turbinate head and anterior to the insertion in two cases (5%), was also above the turbinate insertion,

**Table:** Frequency of the sites where the transillumination point was found on the lateral nasal wall.

	Ant to themth(a)	Post to mth and ant to the mti(b)	Post to the mti	TOTAL
Above mti	.	2	5	7
Immediately below/ mti	7	18	.	25
Below mti	2	6	.	8
TOTAL	9	26	5	40

(a) mth - middle turbinate head

(b) mti - middle turbinate insertion

in eighteen cases (45%) it was immediately below; and in six cases 15%) it was below.

**Figure 4:**
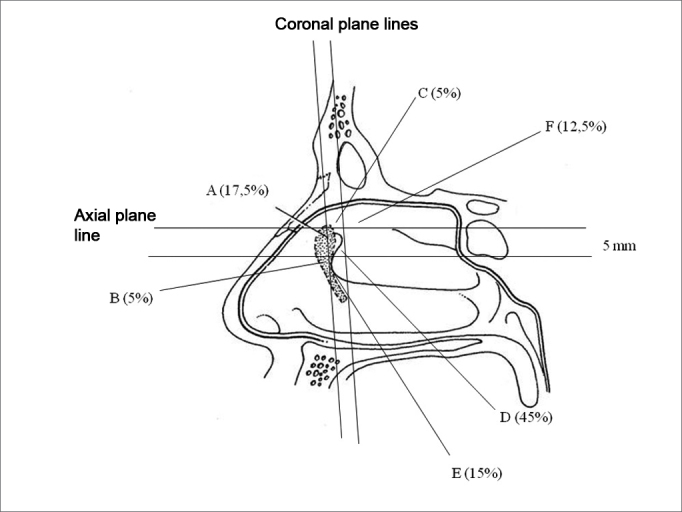
Representative drawing of the sites where the transillumination point was found and its respective frequencies.
AAnterior to the middle turbinate head × immediately below the middle turbinate insertion.BAnterior to the middle turbinate head × below the middle turbinate insertion.CPosterior to the middle turbinate head and anterior to the middle turbinate insertion × above the middle turbinate insertion.DPosterior to the middle turbinate head and anterior to the middle turbinate insertion × immediately below the middle turbinate insertion.EPosterior to the middle turbinate head and anterior to the middle turbinate insertion × below the middle turbinate insertion.FPosterior to the middle turbinate insertion × above the middle turbinate insertion.

The transillumination point, when posterior to the middle turbinate insertion - in five cases (12.5%) it was also above the middle turbinate insertion, in no case it was below the insertion. Figure 4 shows the positions in which the lachrymal sac was found on the lateral nasal wall and how often it was found there.

The maxillary line was prominent in 38 of the 40 lachrymal pathways studied (95%), and it was not present in one cadaver - bilaterally. Nasal septum deviation prevented proper access to the lachrymal sac in five (12.5%) of the 40 lachrymal systems dissected. Removal of the uncinate process was carried out 14 times (35%), and it was bilateral in five cases; only in the right side in two cases and only in the left side in two cases. Middle turbinectomy was carried out in three cases (7.5%); one case to the right and two cases to the left.

## DISCUSSION

DCR-en is a fairly recent procedure when compared to the external approach. The development of the rigid nasal endoscope brought about a new approach to the lachrymal surgery, because it made possible to approach the nasal pathway, avoiding incisions on the face and the very dissection of the orbit orbicular muscle and orbital periosteum^3^,^4^. Notwithstanding, the lachrymal area variability on the lateral nasal wall, as well as its intranasal anatomical parameters and the lack of consensus in the management of these structures translate the very difficulty in standardizing the DCR-en^5^.

The parameters utilized to identify the lachrymal sac on the lateral nasal wall, as well as its management, are described in a variable and inaccurate way in the literature. In general, we know that the anterior portion of the lachrymal fossa is covered by maxillary bone, while the lachrymal bone covers its posterior half and in most of the patients the lachrymal sac is anterior to the free border of the middle turbinate^6^,^7^. Menerath^8^ et al. (1999) and Khoury and Rouvier^9^ use anatomical arguments to base the indication for endonasal DCR, stating that the inferior 2/3 of the lachrymal sac are always in direct association with the nasal fossa in the middle meatus entrance. Met-son^10^ states that the lachrymal sac is located superiorly in the lateral nasal wall and anterior to the middle turbinate. Rebeiz^11^ et al., stated that the lachrymal bone is consistently found anterior to the middle turbinate insertion, and there is very little variation in this location and that its relation with the adjacent nasal structures is constant. In 1989, Mcdonogh and Meiring^6^ described a concave area on the nasal wall, after the nasal vestibule, called atrium, formed by maxillary bone and which has in its posterior portion a vertical elevation which goes from the superior portion of the inferior turbinate all the way to a region immediately anterior to the middle turbinate insertion. This vertical elevation would correspond to the posterior portion of the maxillary bone frontal process. The lachrymal duct and sac would be immediately posterior to the vertical elevation and anterior to the middle turbinate head. Whittet^12^ et al., reported the same relation between this vertical bone elevation, the lachrymal sac and the middle turbinate head. Metson^7^ et al., described the maxillary line as this vertical bony elevation, stressing it as an important parameter for the dissection of the lachrymal sac on the nasal wall. Chastain^13^ et al. described the maxillary line as the key parameter to identify the lachrymal sac on the lateral nasal wall. Yung and Logan^14^ studied the lachrymal bone anatomy on the lateral nasal wall in ten half-heads from human cadavers, and always found it anterior to the middle third of the unciform process and postero-medially covering the superior portion of the lachrymal duct. Wormald^15^ et al., tried to define the lachrymal sac anatomy with high-resolution CT scan images and found it higher in the nasal wall than it is normally described, with its larger portion (10mm) above the middle turbinate and only 1 or 2 mm below this parameter. Cokkeser^16^ et al., in their series with 44 patients submitted to DCR-en, reported that the lachrymal sac was found immediately anterior or under the middle turbinate insertion in all the cases, except in four - when the lachrymal sac was located some millimeters posterior to the turbinate insertion. Komínek^17^ et al. stated that the lachrymal sac is consistently found in the middle turbinate junction with the lateral nasal wall; although it can be found more posteriorly in some cases.

Our attempt to locate the lachrymal sac on the lateral nasal wall was based on the transillumination point of the common canaliculus as an indication of the best site to start the rhinostomy and the relations of this point with nasal anatomical parameters we consider important; and some anatomical considerations can be made.

As previously mentioned, some authors^15,^^18^, ^19^, ^20^ described a lachrymal sac positioned higher on the lateral nasal wall, with its larger portion above the middle turbinate insertion, while others^6^,^12^ indicated that the turbinate insertion was related to the bottom of the lachrymal fossa. These findings show that as an anatomical parameter, the middle turbinate insertion point is still controversial, at least when seen in the coronal plane. Our findings are in agreement with most of the authors^6,^^7,^^12,^^16^ and anatomical descriptions of the lachrymal pathway which report the lachrymal sac bottom some millimeters above the common canaliculus emergence, laterally to the middle turbinate insertion or to the agger nasi. In an antero-posterior direction, the lachrymal sac was more frequently found between the middle turbinate insertion and its free border. Even then the lachrymal sac could be found in almost all the positions described in the literature and transillumination was important to establish the relations between the middle turbinate and the lachrymal fossa. Middle turbinectomy was carried out in 7.5% of the cases in order to improve access to the lachrymal fossa. The decision to resect the middle turbinate was made after the start of dissection so as to make sure of its need. At this point we would also like to stress that transillumination can show a lachrymal sac anterior or superior to an apparently enlarged or hypertrophic middle turbinate, avoiding its resection.

The lachrymal bone is slender and easy to pin point, because it gives immediately to the lateral pressure caused by a curved instrument. Notwithstanding, its endoscopic outline, without using palpation, is not so simple and even to palpate it with an instrument is difficult to distinguish the limits between the lachrymal bone and the unciform process, since they are adjacent structures of similar bone consistencies. Some authors^6,^^7,^^14^ mention the identification of the lachrymal bone joint with the maxillary bone or with the unciform process as an intraoperative parameter. In our material the endoscopic outline of the lachrymal bone, the visualization of the maxillo-lachrymal junction, as well as the lachrymal bone joint with the unciform process was not possible. This may be due to the fact that our dissection was carried out with the zero degree endoscope, which does not provide such a detailed lateral view as the 30 or 45 degree endoscopes.

The maxillary line was a useful and frequent anatomical parameter, being present in almost all our cases (95%); besides being a structure which is easy to identify, visible upon endoscopic inspection, even before the beginning of the dissection. Nonetheless, we agree with Fayet^5^ et al. who reported that it is anterior to the lachrymal sac and both the mucosal incision and the rhinostomy must be carried out posteriorly to this structure. In none of our cases the transillumination point was seen so anteriorly.

Anatomical^14^ and surgical^6^ studies have suggested the unciform process as adjacent and posterior to the lachrymal fossa. Tsirbas and Wormald^19^ stated that such structure must be preserved during DCR-en. Fayet^20^ et al. found some degree of unciform process overlapping the lachrymal fossa in 94.8% of the cases. In our material we deemed uncifectomy necessary in 35% of the dissections. In these cases the optic fiber transilluminated the lachrymal sac and the unciform process, predicting the need for its removal in order to achieve proper exposure of the lachrymal fossa. In all of these cases the transillumination point was posterior to the middle turbinate head or even posterior to its insertion, in a region very near the middle meatus entrance. Notwithstanding, the fact that the transillumination was posterior to the middle turbinate free border or to its insertion, does not necessarily imply in the unciform process removal, which could be explained by the existence of a wide lachrymal bone, providing for a good exposure of the lachrymal fossa without uncifectomy.

The nasal septum deviation made it difficult to access the lachrymal pathway in 12.5% of the cases, always because it was a high deviation, preventing proper access to the lachrymal fossa, not necessarily having a functional impact. The incidence of such procedure during DCR-en is 0.3% to 36% in the literature we studied^7,^^10,^^11,^^16^.

And finally, we would like to stress one aspect of the endoscopic approach to the lachrymal system, which is the overlapping of medical fields: otorhinolaryngology and ophthalmology. The external DCR, described by TOTI1 in 1904, as well as its variations which occurred along the 20th century, have always had in common the fact they were done by ophthalmologists only. The rigid nasal endoscope made this procedure possible through the nose, and today DCR-en is highlighted in the literature^10^,^12^ as a team effort.

## CONCLUSION

• The lachrymal sac was more frequently found underneath the middle turbinate insertion, posterior to its free border and anterior to its insertion.
• However, the procedure standardization based on this location was not possible because of the variable mode with which the lachrymal sac was distributed along the lateral nasal wall, having been found in almost all the positions already described in the literature.
• The common canaliculus transillumination proved useful to identify the lachrymal area, overcoming the anatomical variability issue.
• The common canaliculus transillumination proved useful in locating the lachrymal sac in relation to the middle turbinate, indicating the need to remove or not such structure.
• By the same token, the common canaliculus transillumination proved useful in the possibility of preventing an uncifectomy, showing a lachrymal sac anterior to such structure.
• Septoplasty was necessary whenever we found a high nasal septum deviation preventing access to the lachrymal fossa.
